# Instrument-mounted displays for reducing cognitive load during surgical navigation

**DOI:** 10.1007/s11548-017-1540-6

**Published:** 2017-02-23

**Authors:** Marc Herrlich, Parnian Tavakol, David Black, Dirk Wenig, Christian Rieder, Rainer Malaka, Ron Kikinis

**Affiliations:** 1 Creative Unit: Intra-operative Information, University of Bremen, Bremen, Germany; 2 Serious Games Engineering, University of Kaiserslautern, Kaiserslautern, Germany; 3 Digital Media Lab, TZI, University of Bremen, Bibliothekstr. 1, 28359 Bremen, Germany; 4 Medical Image Computing, University of Bremen, Bremen, Germany; 5 Fraunhofer MEVIS, Bremen, Germany; 6 Surgical Planning Laboratory, Brigham and Women’s Hospital, Boston, USA; 7 Harvard Medical School, Boston, MA USA

**Keywords:** Tool-mounted display, Image-guided surgery, Intra-operative navigation, Visual feedback, Cognitive load, Visual attention

## Abstract

**Purpose:**

Surgical navigation systems rely on a monitor placed in the operating room to relay information. Optimal monitor placement can be challenging in crowded rooms, and it is often not possible to place the monitor directly beside the situs. The operator must split attention between the navigation system and the situs. We present an approach for needle-based interventions to provide navigational feedback directly on the instrument and close to the situs by mounting a small display onto the needle.

**Methods:**

By mounting a small and lightweight smartwatch display directly onto the instrument, we are able to provide navigational guidance close to the situs and directly in the operator’s field of view, thereby reducing the need to switch the focus of view between the situs and the navigation system. We devise a specific variant of the established crosshair metaphor suitable for the very limited screen space. We conduct an empirical user study comparing our approach to using a monitor and a combination of both.

**Results:**

Results from the empirical user study show significant benefits for cognitive load, user preference, and general usability for the instrument-mounted display, while achieving the same level of performance in terms of time and accuracy compared to using a monitor.

**Conclusion:**

We successfully demonstrate the feasibility of our approach and potential benefits. With ongoing technological advancements, instrument-mounted displays might complement standard monitor setups for surgical navigation in order to lower cognitive demands and for improved usability of such systems.

## Introduction

Surgical navigation systems are becoming more common throughout different disciplines, often used for operations and interventions involving very delicate structures, structures that are not perceivable without medical imagery, and for minimally invasive interventions where the natural field of view of the operator is limited.

This work focuses on needle-based interventions. However, our approach could generalize to many procedures involving an instrument large enough to carry a small display. In RF ablation and comparable procedures, a needle is inserted into the body and the tip is navigated to the target structure, such as a liver tumor, which is ablated by applying heat caused by electric current or microwaves. Our work concentrates on navigation; intervention and treatment are not considered. As the remainder of this paper will describe in more detail, the current prototype is based on a phantom created for RF ablation as a testbed, which is usually performed by interventional radiologists. However, the concept and implementation could in principle be applied more broadly also to surgical procedures or for educational purposes that involve the guidance of needles or other instruments that are inserted at a specific location and angle into the patient’s body.

The challenge is to provide intuitive visual feedback during a procedure. Typically, a monitor is placed either in front or to the side of the operator. Placing the monitor can be quite challenging and subject to many constraints, such as limited space and line of sight. The monitor should be placed near the operator but without limiting the movements. Therefore, the operator has to divide the visual attention between situs and display. This is generally not desirable, as it could increase cognitive load, slow the procedure, and possibly reduce accuracy.

We propose attaching a small display to the instrument to provide navigational guidance. A small display does not occlude the field of vision, is light enough to place directly onto the instrument without putting additional mechanical strain on the operator, and reduces the need of splitting the visual attention. While others have proposed comparable approaches, to the best of our knowledge, these involve larger displays, head-mounted displays (HMDs) or complex projection setups. Using small displays is not simply a matter of absolute size, but of tailoring a visualization to the small screen.

We conducted an empirical study with 25 participants to investigate the benefits of our approach. We compared three conditions: using only the small instrument-mounted display (IMD), using the IMD with a monitor and using only a monitor (control condition). Our results show significant differences in favor of our approach regarding cognitive load, user preference and usability, while task completion times and overall accuracy are comparable across all conditions. Additionally, we found significant differences in favor of the IMD for normalized view percentages for the combined condition.

## Related work

With the increasing interest of researchers and practitioners in surgical navigation over the last decades, the topic has become very broad and deep. Therefore, we limit the discussion to approaches most similar to our own work. We refer the reader to Mezger et al. [13] for a more general review of surgical navigation systems and to Mundeleer et al. [14] for an example of a system developed specifically for RF ablation.

Employing augmented (AR) and mixed reality (MR) to overlay situs and structures with navigational information has been investigated by a number of researchers in the past. Existing works can be divided into approaches that use HMDs [1, 3, 16] to overlay the information onto the natural field of view, approaches that feed the information into microscopes [6] or video streams [7, 10], and approaches using projectors [8].

In general, AR and MR systems fuse navigational information with the real view of the situs and organs as seen by the operator, reducing the information mapping problem. HMD and projection approaches require no monitor and solve the problem of splitting the visual attention. However, HMDs heavily instrument the operator and might be incompatible with other equipment or glasses. Projections suffer from difficult lighting conditions inside the OR.

Other researchers proposed mounting displays or devices with displays directly onto tools and instruments [2, 5, 12] (as do we) or use mirrors to achieve a similar effect [15]. While we share the principal motivation, this work differs in many important aspects from existing solutions. We investigate a different form factor, and without empirical validation it remains unclear whether such a small display will be able to provide the navigational information.

We focus on cognitive load and needle-based interventions, in contrast to Kassil and Stewart [12], who focus on performance and drilling tasks.

The Brainlab Dash is a commercially available solution that allows to mount an iPod onto a range of surgical instruments that have been equipped with a matching mount. It has been employed and studied successfully for knee and hip replacements, as discussed by Bäthis [2]. Although the Brainlab Dash also provides an IMD, it employs the much larger iPod at the cost of added weight and bulkiness, which is used to provide an interface very similar to what is normally available on the standard monitor. In contrast, our work employs a much smaller display with the advantage of much reduced weight and bulkiness but the need for a more focused visualization. Furthermore, to the best of our knowledge, the Dash has not been studied with regard to the application area and cognitive measures we investigate.

The intelligent wielding gun proposed by Echtler et al. [5] uses similar visual feedback to guide workers in car manufacturing; however, the form factor and application area are quite different. The wielding gun is intended to find target locations on a surface and not within a body or structure, and it was not evaluated in a controlled laboratory setting with respect to cognitive load. Stetten and Chib [15] provide ultrasound images and not navigational information. To the best of our knowledge, benefits in terms of cognitive load and physical demand have not been empirically validated in our specific scenario.

**Fig. 1:**
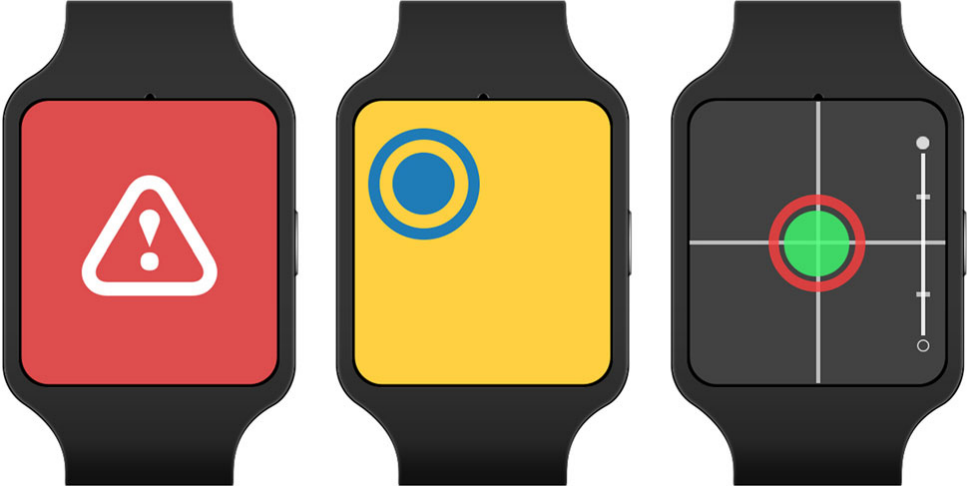
Modes indicating different states (from *left* to *right*): tool outside of tracking range, tool far away from insertion point, tool in close enough distance to insertion point

Auditory displays [9] have been investigated to reduce reliance on an external monitor. However, current approaches rely on serializing a navigation task into several sequential sub-tasks and might not scale well for complex navigational tasks. They also suffer from problems inside a crowded OR. Still, these approaches are promising and may complement our approach in the future.

## Concept and implementation

Variants of the crosshair metaphor are established in navigation for guidance, in addition to presenting 3D images from different perspectives [2, 13]. Using 2D visual guides for the 3D task of inserting a needle at the right point with the correct angle and depth may be easier and quicker to understand than relying solely on the complex 3D information [1]. Currently, most systems present both information types, which is not possible on a small display. One goal of this work is to empirically validate complete reliance on the abstracted 2D guides. We designed a crosshair metaphor that maps the shaft and the tip of the needle onto two colored circles. By moving the instrument until both are centered on the display, the needle is navigated into the correct position and orientation. A scaled bar is filled during insertion to indicate the correct depth.

We introduced clearly separated modes to indicate additional states: a failure mode when the needle is outside the tracking area, a targeting mode if the needle is far from the starting point, and the navigation mode as described above (Fig. 1).

**Fig. 2:**
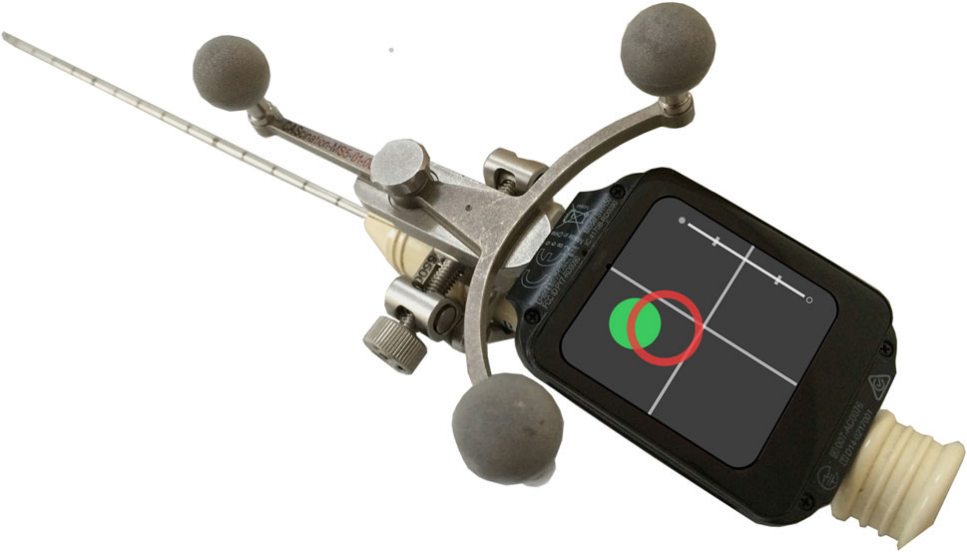
Picture of the prototype as used in the experiment with the small display mounted directly on the needle

In targeting mode, the crosshair is not displayed, as the small distance shown on the display might be misleading and different scaling factors are used on the real needle distance to display the circle representing the needle within the bounds of the small display. Thus, the relative direction with respect to the display center is preserved but not the distance. The user is guided toward the correct location by bringing the blue circle closer to the center, and at a fixed threshold that is dependent on the navigation system and display size, the screen will switch to navigation mode. The application we used for this work maps the 3D location of the needle’s tip and shaft finally to points provided in 2D screen coordinates. For maximum precision, the switch to navigation mode happens when distances can be presented at pixel accuracy, which corresponds to using the size of the small display measured in pixels as threshold for switching modes.

**Fig. 3:**
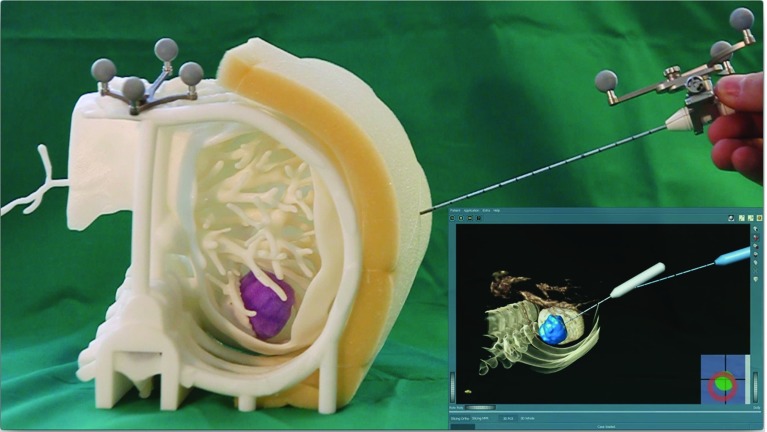
Picture of the foam-based phantom and the look of the RF ablation application used on the monitor. The planned (*gray*) and the tracked needle (*blue*) are displayed in a 3D view and a crosshair based 2D guide in the *bottom right*

We implemented our prototype as an Android Wear application on a Sony Smartwatch 3 with a 1.6-in $320\times 320$ pixel display (Fig. 2). The dimensions of the display module are $50\times 35\times 10$ mm (height $\times$ width $\times$ thickness), and it weighs 39 g. We used a CAS-One^1^ optical surgical navigation system. An application developed for RF ablation procedures provided the test environment for our studies (Fig. 3). The application presents 3D views and a 2D crosshair guide similar to state-of-the-art systems. The application sends navigational information over Wi-Fi as OSC^2^ packages to the smartwatch display.

## User study

We performed a controlled experiment to evaluate against the current state of the art: a standard monitor setup for image-guided surgery. We hypothesized that a small IMD:

- **H1** Can lead to successful navigation.
- **H2** Reduces the cognitive load on surgeons.
- **H3** Reduces time looking at an external monitor.
- **H4** Increases the overall usability of the navigation system.
- **H5** Improves the performance (time/accuracy) of the procedure.

For the experiment, we used a 3D-printed phantom of a patient’s liver including ribs, vessels, and tumor extracted from a contrast enhanced CT dataset (Fig. 3) and affixed on a table in front of the participants. Foam attached to the phantom provided tactile resistance. The tracking unit and monitor was placed on the right side.

We ensured that participants coming from a non-medical background understood the task. They were asked to stand in front of the phantom and grasp the needle with their dominant hand. The task consisted of placing the needle at the correct entry point and inserting it at the correct angle to the correct depth. The participants were instructed to follow the optimal path as precisely as possible and bring the tip of the needle into the correct position in the middle of a virtual target tumor. The experiment used a within-subjects design including three conditions, which differed only in the way the navigational information was presented:

- **C1** Only on the small IMD
- **C2** Simultaneously on the IMD and on a monitor
- **C3** Only on a monitor (control condition)

The order of conditions was pseudo-randomized across participants using a latin-square scheme to counterbalance for potential learning effects. The IMD remained attached to the needle for all conditions but was turned off for C3. For each condition, participants trained the task at least once without recording any data until they felt confident enough to proceed. During the test, participants performed three repetitions of the task for each condition. They were given a maximum time limit of 3 min to complete each repetition, which were considered complete when the participants announced they had reached the best position according to the navigational information. If the repetition was not completed within the time limit, they were asked to stop the repetition and start the next. We recorded the position and orientation of the instrument and task completion time. After each condition, the participants completed a short questionnaire as described below. Participants were informed they could and should ask for a break if needed, however, the short breaks while filling out the questionnaires and the instructor preparing the next condition provided enough time for resting as no participant asked for an additional break. All sessions were recorded on video for further analysis. A complete session (all three conditions) took approximately 40 min on average.

The questionnaire included the NASA-TLX [11] for measuring cognitive load and the System Usability Scale (SUS) [4] for overall usability. We added a question (user preference) concerning overall preference on a five-point Likert scale. Accuracy was calculated using dynamic time warping (DTW) and calculating the deviation from the optimal path defined as a polygon from the start at the insertion point to the destination point. Using post-experimental video analysis, we manually measured the time percentages for participants view at the small screen versus the monitor during C2 (combined condition). Completion times and accuracy were averaged across the three task repetitions for each condition.

## Results

Twenty-five users (10 female/15 male), all students from different fields, with an average age of 28.84 years (SD$\,=\,4.15$) participated. None suffered from physical disabilities. Nineteen participants had mild viewing disabilities and wore their glasses during the experiment. None of the participants were familiar with medical navigation systems, while eight had prior-experience using smartwatches.

The means and standard deviations for cognitive load, user preference, usability, task completion time, and accuracy are summarized in Table 1.

**Table 1:** Means ± standard deviations across all three conditions ($n = 25$) for cognitive load (NASA-TLX; [0, 100]; lower is better), user preference (Likert scale; [1, 5]; higher is better), usability (SUS; [0, 100]; higher is better), task time (s), and accuracy (mm)

	C1 (IMD)	C2 (combined)	C3 (monitor)
Cognitive load	$27.3 \pm 13.85$	$29.37 \pm 14.03$	$37.6 \pm 14.91$
User preference	$4.44 \pm 0.65$	$4.56 \pm 0.71$	$3.6 \pm 1.08$
Usability	$82.2 \pm 12.96$	$78.2 \pm 14.35$	$71.0 \pm 14.51$
Task time	$39.63 \pm 28.41$	$44.43 \pm 29.87$	$46.36 \pm 26.09$
Accuracy	$20.42 \pm 3.36$	$20.29 \pm 3.84$	$22.01 \pm 4.12$

Cognitive load was calculated as the overall task load index according to the NASA-TLX questionnaire, which results in an overall score ranging between 0 (best) and 100 (worst). User preference was calculated as the result of a custom question rated on a 5-point Likert scale, where higher means better. Usability is reported as the overall score according to the SUS questionnaire, which results in a score ranging between 0 (worst) and 100 (best).

We conducted a one-way repeated-measures analysis of variances (RM-ANOVA) for all measures and checked for contrasts. We used Mauchly’s test to check the RM-ANOVA pre-condition of sphericity. In cases that violate the sphericity assumption, we report the Greenhouse–Geisser-corrected results. If indicated by significant results of the RM-ANOVA, we used Sidak-corrected *t* tests for dependent groups to do a full set of pairwise post hoc comparisons. We used the statistical package SPSS (v23), which factors the Sidak correction directly into the *p* value, which therefore should be compared against the uncorrected alpha level of 0.05 to check for statistical significance.

Mauchly’s test did not show a violation of sphericity (W(2) $=$ 0.97, $p=0.707$) for cognitive load. RM-ANOVA revealed a highly significant difference across all conditions ($F(2,48)=5.826, p=0.005$, partial $\eta ^{2}=0.195$) as well as a highly significant linear contrast ($F(1,24)=11.874, p=0.002$, partial $\eta ^{2}=0.331$). Pairwise comparison revealed a highly significant difference between C1 and C3 ($p=0.006$) and a trend for a difference between C2 and C3 ($p=0.074$) but no significant difference between C1 and C2.

For user preference, Mauchly’s test did not show a violation of sphericity ($W(2)=0.812, p=0.091$). RM-ANOVA revealed a significant difference across all conditions ($F(2,48)=14.710, p<0.001$, partial $\eta ^{2}=0.380$) as well as a highly significant linear contrast ($F(1,24)=20.510, p<0.001$, partial $\eta ^{2}=0.461$) and a significant quadratic contrast ($F(1,24)=5.864, p=0.023$, partial $\eta ^{2}=0.196$). Pairwise comparison revealed a highly significant difference between C1 and C3 ($p=0.002$) and C2 and C3 (${p}<0.001$) but no significant difference between C1 and C2.

Mauchly’s test revealed a violation of sphericity for usability ($W(2)=0.769, p=0.049$). RM-ANOVA with Greenhouse–Geisser correction ($\epsilon =0.812$) revealed a highly significant difference across all conditions ($F(1.625,38.991)=8.295, p=0.002$, partial $\eta ^{2}=0.257$) as well as a highly significant linear contrast ($F(1,24)=11.488, p=0.002$, partial $\eta ^{2}=0.324$). Pairwise comparison revealed a highly significant difference between C1 and C3 ($p=0.007$), a strong trend for a difference between C2 and C3 ($p=0.051$) but no significant difference between C1 and C2.

RM-ANOVA revealed no significant differences across conditions for time and accuracy, and consequently, pairwise comparisons were not conducted.

A one-sample *t* test against the 50% level (assumed equal view distribution) for the average normalized view percentage measured in C2 for the small display ($M=67.385$, SD $=$ 29.6) and the monitor ($M=32.615$, SD $=$ 29.6) revealed a highly significant difference ($t(23)=2.877, p=0.009$).

## Discussion

The results strongly support our main goals and hypotheses: Providing navigational information on an IMD was possible (H1) and significantly reduced the cognitive load as measured by the NASA-TLX (H2). We attribute this to two main factors: first, our setup eliminates or significantly reduces dividing visual attention between situs and monitor. Second, for situations where switching the view between the situs and monitor requires head movement, physical stress is reduced by the IMD. These benefits are reflected by significant differences for user preference and overall usability in favor of our approach (H4). As shown by the significant differences of the normalized view percentages, participants strongly favored the IMD even when a monitor was present (H3). This is supported by the pairwise comparisons where significant differences were clearly revealed between C1 (IMD) and C3 (external monitor) and, to a lesser extent, between C2 (combined condition) and C3 (external monitor), while we found no significant differences between C1 and C2.

Related work [12] suggests that an IMD might benefit objective user performance. Yet, our analysis did not reveal any significant differences in time and accuracy across conditions (H5). Looking at the absolute numbers, there are slight differences in favor of our approach. However, these cannot be distinguished from statistical noise. This suggests that the effect (should it be present) is at least not as strong as the effect of reducing the cognitive load. This could be a due to feedback design, which may not be optimal in that regard, or a general limitation of the small display space compared to the bigger displays used in related works, which might better provide feedback on accuracy. Having achieved comparable user performance across conditions means that our feedback design successfully communicated the navigational information at least as well as the control condition, which was not immediately clear before gathering empirical data and thus is an important contribution in itself.

Although the results strongly show the benefits of the IMD, there are some limitations. First, we chose to place the monitor to the side of the participants rather than directly in front, as we had witnessed in clinical practice. This could be regarded as a worst-case situation by some and in fact there are OR setups where the monitor is directly in front of the operator. We argue that while this might lead to better absolute values for the control condition, the monitor can never be placed next to the situs. Our principal arguments and results should also be valid for this best-case situation, although effect size might slightly decrease.

A second limitation is the inclusion of only participants without a medical background. In comparison with medical experts, we would expect the absolute numbers to be different and to depend on the specific procedure. For instance, expert interventional radiologists might only need a couple of seconds to place the needle in simple cases. However, the relative benefits should in principle be applicable also to expert users and based on the positive indications of this work, conducting more extensive studies with expert users seems to be justified. The benefits of reduced cognitive load will naturally be more pronounced the more complex and time-consuming the procedure; however, even small benefits for comparatively short procedures might add up if conducting many of these procedures.

The current prototype would not fulfil the constraints of a real OR in terms of hygiene and electrical safety; however, our goal is to collect evidence on the principal approach as we believe that the display technology can be produced according to those constraints.

## Conclusion

We presented a concept and prototype for providing navigational information through a small display mounted directly on the instrument. Our approach contributes to the current state of the art by showing how to utilize limited screen size successfully. This approach offers several benefits as the display is small enough to not occlude the situs or strain the instrument or operator while significantly reducing cognitive load caused by dividing visual attention with an external monitor. We provide empirical data from a user study with 25 users comparing three conditions, i.e., the IMD, a combination of IMD and external monitor, and only using an external monitor. This revealed significant differences in favor of our approach for cognitive load, user preference, and general usability while achieving comparable objective performance measures in terms of time and accuracy across all conditions. Although participants came from a non-medical background, the task was easy enough for non-medical experts. We expect no principal performance differences between novices and experts across conditions. Future work will extend the study to medical experts.

## Footnotes

1 CAScination AG, Switzerland.

2 Open Sound Control.
